# Pervasive, Genome-Wide Transcription in the Organelle Genomes of Diverse Plastid-Bearing Protists

**DOI:** 10.1534/g3.117.300290

**Published:** 2017-09-20

**Authors:** Matheus Sanitá Lima, David Roy Smith

**Affiliations:** Department of Biology, University of Western Ontario, London, Ontario N6A 5B7, Canada

**Keywords:** RNA-seq, mitochondrial transcription, organelle gene expression, plastid transcription, protists

## Abstract

Organelle genomes are among the most sequenced kinds of chromosome. This is largely because they are small and widely used in molecular studies, but also because next-generation sequencing technologies made sequencing easier, faster, and cheaper. However, studies of organelle RNA have not kept pace with those of DNA, despite huge amounts of freely available eukaryotic RNA-sequencing (RNA-seq) data. Little is known about organelle transcription in nonmodel species, and most of the available eukaryotic RNA-seq data have not been mined for organelle transcripts. Here, we use publicly available RNA-seq experiments to investigate organelle transcription in 30 diverse plastid-bearing protists with varying organelle genomic architectures. Mapping RNA-seq data to organelle genomes revealed pervasive, genome-wide transcription, regardless of the taxonomic grouping, gene organization, or noncoding content. For every species analyzed, transcripts covered ≥85% of the mitochondrial and/or plastid genomes (all of which were ≤105 kb), indicating that most of the organelle DNA—coding and noncoding—is transcriptionally active. These results follow earlier studies of model species showing that organellar transcription is coupled and ubiquitous across the genome, requiring significant downstream processing of polycistronic transcripts. Our findings suggest that noncoding organelle DNA can be transcriptionally active, raising questions about the underlying function of these transcripts and underscoring the utility of publicly available RNA-seq data for recovering complete genome sequences. If pervasive transcription is also found in bigger organelle genomes (>105 kb) and across a broader range of eukaryotes, this could indicate that noncoding organelle RNAs are regulating fundamental processes within eukaryotic cells.

Mitochondrial and plastid DNAs (mtDNA and ptDNAs) are among the most sequenced and best-studied types of chromosome (Smith 2016b). This is not surprising given the widespread use of organelle genome data in forensics, archeology, phylogenetics, biotechnology, medicine, and other scientific disciplines. Unfortunately, investigations of organelle RNA have not kept pace with those of the DNA, and for most nonmodel species there are little or no published data on organelle transcription (Sanitá Lima *et al.* 2016). But this is poised to change.

Next-generation sequencing (NGS) technologies, ballooning genetic databanks, and new bioinformatics tools have made it easier, faster, and cheaper to sequence, assemble, and analyze organelle transcriptomes (Smith 2016b). The National Center for Biotechnology Information (NCBI) Sequence Read Archive (SRA), for example, currently houses tens of thousands of freely available eukaryotic RNA sequencing (RNA-seq) datasets (Kodama *et al.* 2012), hundreds of which come from nonmodel species and/or poorly studied lineages (Keeling *et al.* 2014). Among their many uses, these data have proven to be a goldmine for mitochondrial and plastid transcripts (Smith 2013; Shi *et al.* 2016; Tian and Smith 2016).

Recently, researchers have started mining the SRA for organelle-derived reads, and already these efforts have yielded interesting results, such as pervasive organelle transcription, *i.e.*, transcription of the entire organelle genome, including coding and noncoding regions (Shi *et al.* 2016; Tian and Smith 2016). This kind of research has been further aided by a range of new bioinformatics tools designed for the assembly, annotation, and analysis of organelle genomes and transcriptomes from NGS data (Castandet *et al.* 2016; Dierckxsens *et al.* 2016; Soorni *et al.* 2017). Nevertheless, most of the eukaryotic RNA-seq data within the SRA have not been surveyed for organelle transcripts, particularly those from plastid-bearing protists, and it is not known if pervasive organelle transcription is a common theme among diverse eukaryotic groups. If it is, then RNA-seq could presumably be used to glean complete or near-complete organelle genomes in the presence or absence of DNA data, which would be particularly useful, for example, in cases where there are abundant RNA-seq data but no available DNA information.

It goes without saying that the complexities of organelle transcription cannot be unraveled solely via *in silico* RNA-seq analyses (Sanitá Lima *et al.* 2016). Indeed, organelle gene expression is surprisingly complex and often highly convoluted (Moreira *et al.* 2012), as anyone who has studied the mtDNA of *Trypanosoma* spp. (Feagin *et al.* 1988) or the ptDNA of *Euglena gracilis* (Copertino *et al.* 1991) can attest. If organelle transcriptional research has taught us anything over the past few decades, it is that even the seemingly simplest mtDNAs and ptDNAs can have unexpectedly complicated transcriptomes and/or modes of gene expression (Feagin *et al.* 1988; Copertino *et al.* 1991; Marande and Burger 2007; Masuda *et al.* 2010; Vlcek *et al.* 2011; Lang *et al.* 2014; Valach *et al.* 2014; Smith and Keeling 2016). Moreover, accurately and thoroughly characterizing organelle transcriptional architecture can take years of detailed laboratory work using an assortment of techniques (Marande *et al.* 2005; Nash *et al.* 2007; Barbrook *et al.* 2012; Feagin *et al.* 2012; Jackson *et al.* 2012; Mungpakdee *et al.* 2014; Dorrell and Howe 2015). That said, RNA-seq is a quick and cost-effective starting point for early exploratory work of organelle transcription, and it can help identify lineages or species with particularly bizarre or unconventional transcriptional architectures.

Here, we use publicly available RNA-seq data to survey mitochondrial and plastid transcription in a variety of eukaryotic algae. To streamline and simplify our analyses, we focus specifically on species for which the mitochondrial and/or plastid genomes have been completely sequenced and are not overly long (≤105 kb). Our explorations reveal pervasive, genome-wide organelle transcription among disparate plastid-bearing protists and highlight the potential of freely available RNA-seq data for organelle research.

## Materials and Methods

By scanning the SRA (using NCBI’s Taxonomy Browser), we identified 30 plastid-bearing species for which there are complete mitochondrial and/or plastid genome sequences and abundant RNA-seq data. We downloaded the RNA-seq reads from the SRA (https://www.ncbi.nlm.nih.gov/sra) and the organelle DNAs from the Organelle Genome Resources section of NCBI (https://www.ncbi.nlm.nih.gov/genome/organelle/) or GenBank (https://www.ncbi.nlm.nih.gov/genbank/). See Supplemental Material, Table S1 for detailed information on the RNA-seq and organelle genome data we downloaded, including accession numbers, sequencing technologies, read counts, organelle DNA features, and the strains used for genome sequencing and RNA-seq.

We mapped the RNA-seq reads to the corresponding organelle genomes using Bowtie 2 (Langmead and Salzberg 2012) implemented through Geneious v9.1.6 (Biomatters Ltd., Auckland, NZ), a user-friendly, commercial bioinformatics software suite, which contains a graphical user interface (Kearse *et al.* 2012). All mapping experiments were carried out using default settings, the highest sensitivity option, and a min/max insert size of 50 nt/750 nt; we also allowed each read to be mapped to two locations to account for repeated regions, which are common in organelle genomes (Smith and Keeling 2015). The mapping histograms shown in Figure 2, Figure 3, and Figure 4 were extracted from Geneious.

### Data availability

The datasets analyzed in this study are available in the SRA database (https://www.ncbi.nlm.nih.gov/sra/) and their respective accession numbers are listed in Table S1. Figure S1 depicts transcription maps for all 30 species analyzed.

## Results and Discussion

### Little genome, big RNA: genome-wide, polycistronic transcription in algal organelle DNAs

**Figure 1:**
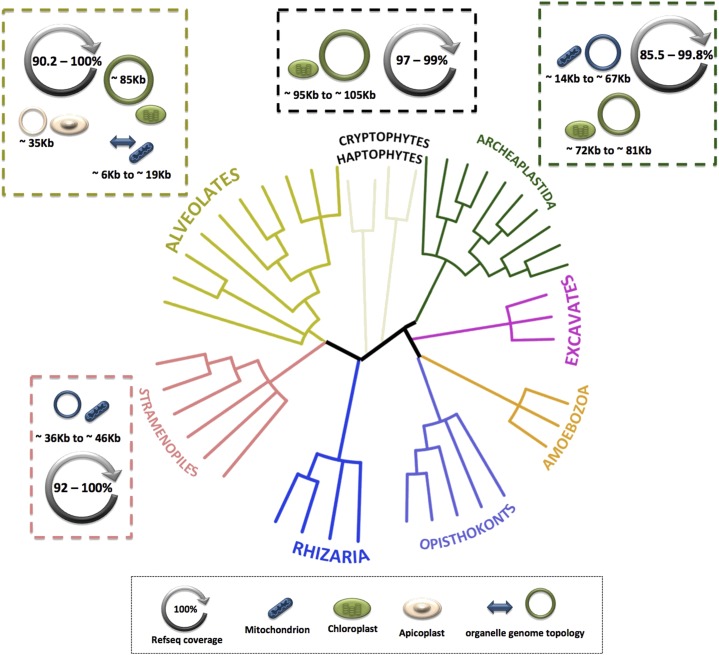
Pervasive organelle genome transcription across the eukaryotic tree of life. Organelle genomes ≤105 kb are fully or almost fully transcribed in diverse eukaryotic groups, regardless of their coding content and structure. Outer dashed boxes summarize the breadth of organelle genomes analyzed within each major eukaryotic group. Representation of organelle genomes and organelles are not to scale. Refseq coverage represents the percentage of the reference genome sequence that was covered by one or more RNA-seq reads in the mapping analyses. Phylogenetic tree is adapted from Burki (2014) for the relationships among major groups; branches within groups are merely illustrative and not based on sequence analyses. The tree was generated using the NCBI Common Tree taxonomy tool (Federhen 2012) and iTOL v3.4.3 (Letunic and Bork 2016).

**Figure 2:**
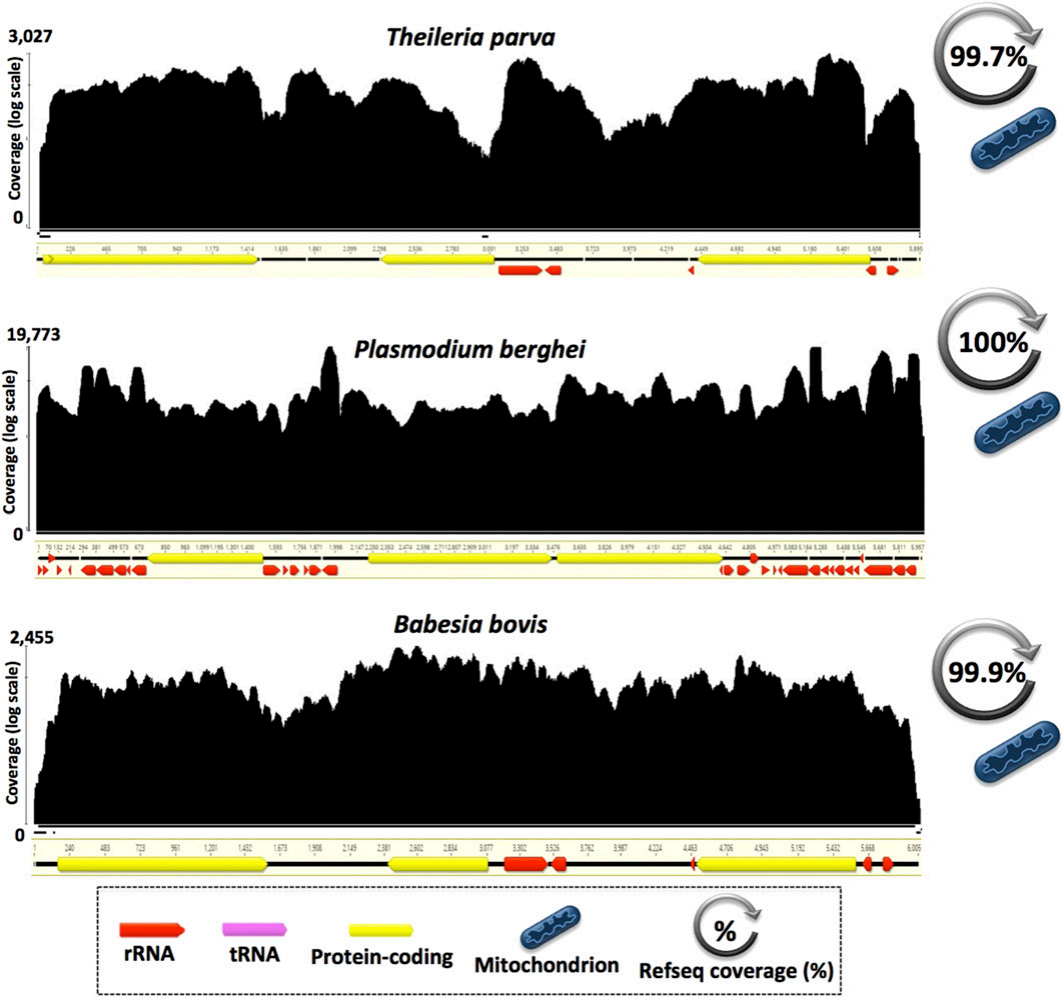
Full transcription of small mitochondrial genomes in Apicomplexa. Mapping histograms (or transcription maps) depict the coverage depth—number of transcripts mapped per nucleotide—on a log scale. We used the organelle genome annotations already present in the genome assemblies deposited in GenBank (accession numbers provided in Table 1 and Table S1). Mapping contigs are not to scale and direction of transcription is represented by the direction of the arrows: annotated genes. Mapping histograms were obtained from Geneious v9.1.6 (Kearse *et al.* 2012).

**Figure 3:**
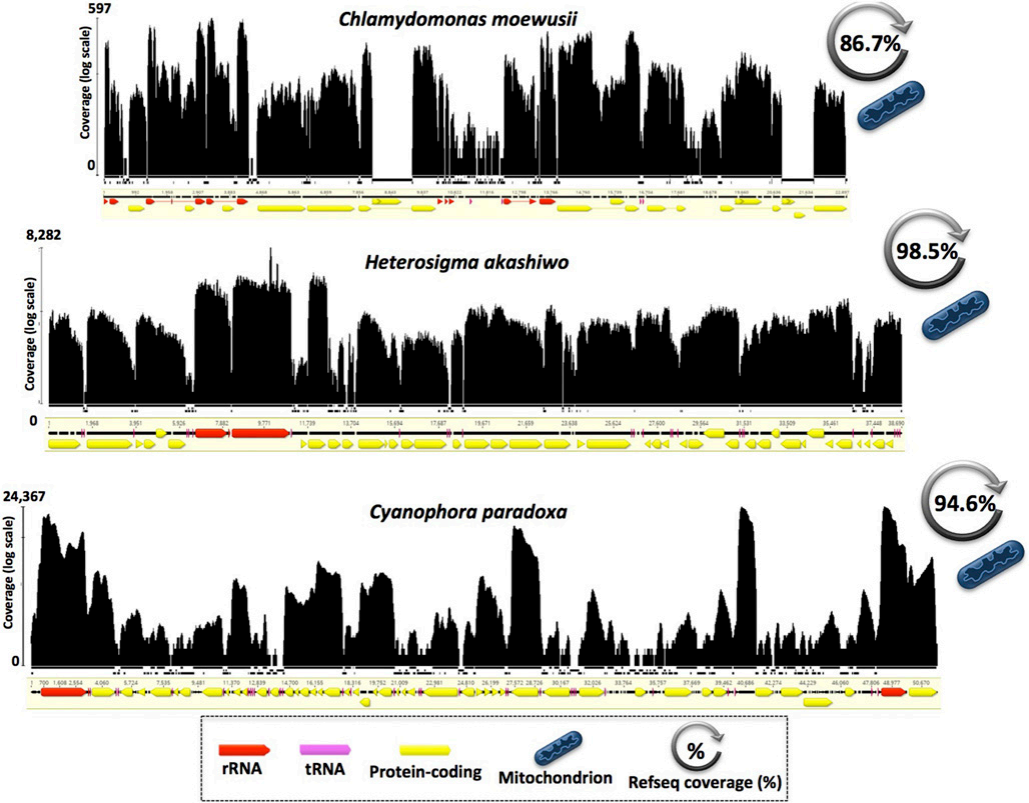
Polycistronic transcription in mitochondrial genomes of chlorophytes, raphidophytes, and glaucophytes. *C. moewusii* (Chlorophyta), *H. akashiwo* (Raphidophyta), and *C. paradoxa* (Glaucophyta) exhibited clear drops of transcript coverage in some potentially noncoding regions (intergenic regions, introns, and hypothetical proteins). Mapping histograms follow the same structure as in Figure 2 and mapping contigs are not to scale.

**Figure 4:**
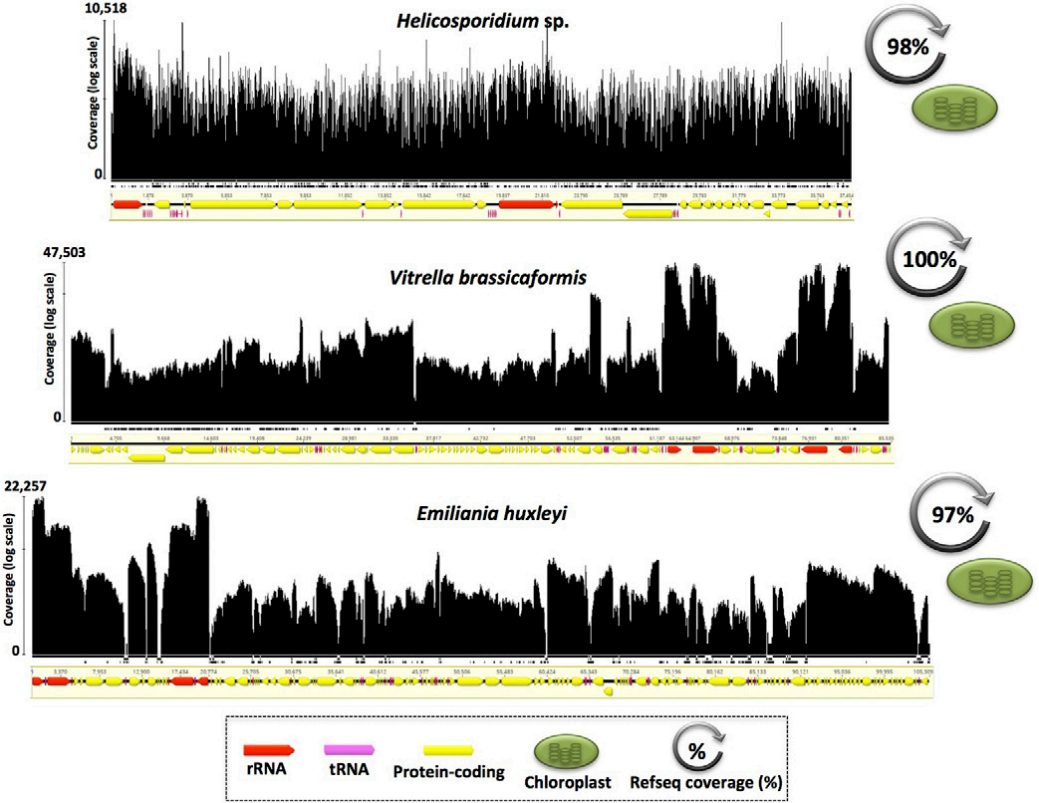
Entire and near entire transcriptional coverage of diverse plastid genomes. *V. brassicaformis* (Chromerida) exhibited entire genome transcription, whereas *Helicosporidium* sp. (Chlorophyta) and *E. huxleyi* (Haptophyta) had near entire genome transcriptional coverage. Drops in coverage happened mostly in intergenic regions of the *E. huxleyi* plastid genome. Mapping histograms follow the same structure as in Figure 2 and Figure 3; mapping contigs are not to scale.

After an exhaustive search of GenBank and the SRA, we identified 30 plastid-bearing protists for which there were abundant RNA-seq data as well as complete mtDNA and/or ptDNA sequences with lengths of ∼100 kb or smaller. We did not include larger organelle DNAs because we wanted to reconstruct entire organelle genomes from the transcript data alone and assumed that it would be easier to do so using RNA from small to moderately sized organelle genomes. Moreover, organelle DNAs >100 kb are typically repeat rich (Smith and Keeling 2015), making RNA-seq mapping much more challenging and error-prone (Treangen and Salzberg 2011). Nonetheless, the 30 species we analyzed span the gamut of plastid-containing eukaryotic diversity, and include taxa with primary plastids and eukaryote-eukaryote-derived (*i.e.*, “complex”) plastids (Keeling 2013) as well as those with DNA-containing nonphotosynthetic plastids, such as apicomplexan parasites (Figure 1, Figure S1, Table 1, and Table S1). The organelle genomic architectures of these species vary in structure (*e.g.*, linear- *vs.* circular-mapping), size (5.8–105 kb), gene repertoire (*e.g.*, gene rich *vs.* gene poor), gene arrangement (*e.g.*, intact *vs.* fragmented genes), and coding content (*e.g.*, ∼7.5–95%) (Figure 2, Figure 3, Figure 4, Figure S1, Table 1, and Table S1). We made sure that the RNA-seq and corresponding organelle genome data always came from the same species, but, in a few instances, they were from different strains of the same species (Table S1). It should be stressed that most of the RNA-seq experiments we sourced were generated under stress-related conditions and often using very different protocols (Table S1). But these caveats did not seem to impede the mapping experiments.

**Table 1:** Diverse organelle (mitochondrial and plastid) genomes and their respective transcription rates (mean and percent coverage)

Taxonomic Group and Species	Organelle	GenBank Entry	Genome Size (bp)	Mean Coverage (Reads/nt)	% Refseq*^a^*	% Coding*^b^*
API - *Theileria parva*	MT	NC_011005.1	5,895	710.9	99.7	67.5
API - *Plasmodium berghei*	MT	LK023131.1	5,957	3,111.9	100	92.4
API - *Plasmodium falciparum*	MT	AY282930.1	5,959	368.3	100	55.7
API - *Plasmodium vivax*	MT	NC_007243.1	5,990	693.6	100	56.3
API - *Babesia bovis*	MT	NC_009902.1	6,005	614.8	99.9	63.5
	APIC	NC_011395.1	35,107	71.6	90.2	54.1
API - *Babesia microti*	MT	LN871600.1	10,547	5.2	93.4	37
CP - *Chlamydomonas leiostraca*	MT	NC_026573.1	14,029	136.9	95.8	86.4
DF - *Symbiodinium minutum*	MT	LC002801	19,577	2,763	100	7.4
CP - *Chlamydomonas moewusii*	MT	NC_001872.1	22,897	59.8	86.7	55.4
CP - *Pycnococcus provasolii*	MT	GQ497137	24,321	2,942.4	99.8	87.7
PP - *Fucus vesiculosus*	MT	NC_007683.1	36,392	98.9	97.9	90
RP - *Porphyra purpurea*	MT	NC_002007.1	36,753	1,250.4	98.7	81.5
RP - *Pyropia haitanensis*	MT	NC_017751.1	37,023	24.4	85.6	63.2
PP - *Undaria pinnatifida*	MT	NC_023354.1	37,402	165.1	92.8	89.9
PP *- Saccharina japonica*	MT	NC_013476.1	37,657	145.9	100	89.4
EP - *Nannochloropsis oceanica*	MT	NC_022258.1	38,057	118.7	95.8	88.8
RH - *Heterosigma akashiwo*	MT	NC_016738.1	38,690	205.2	98.5	81.3
RP - *Pyropia yezoensis*	MT	NC_017837.1	41,688	16.2	88	56.6
DT - *Pseudo-nitzschia multiseries*	MT	NC_027265.1	46,283	1,261.3	96.4	71.5
CP - *Micromonas commoda*	MT	NC_012643.1	47,425	180.6	94	82.5
CP - *Helicosporidium* sp.	MT	NC_017841.1	49,343	147.4	94.7	65
	PT	NC_008100.1	37,454	103.6	98	94.9
GP - *Cyanophora paradoxa*	MT	NC_017836.1	51,557	3,355.9	94.6	58.9
CP - *Chlorella sorokiniana*	MT	NC_024626.1	52,528	23,494.2	86.6	63
CA - *Chara vulgaris*	MT	NC_005255.1	67,737	24.9	94.2	52.3
CP - *Micromonas commoda*	PT	NC_012575.1	72,585	2,854.1	93.7	67.8
CP - *Picocystis salinarum*	PT	NC_024828.1	81,133	142.1	85.5	90.6
CR - *Vitrella brassicaformis*	PT	HM222968	85,535	5,523.6	100	88.5
HP - *Emiliana huxleyi*	PT	NC_007288.1	105,309	789.9	97	85.8
HP - *Pavlova lutheri*	PT	NC_020371.1	95,281	2,771.8	99.4	81
API - *Toxoplasma gondii*	APIC	NC_001799.1	34,996	1,501.4	95	80.7

API, Apicomplexa; MT, mitochondrion; CP, Chlorophyta; DF, Dinoflagellates; PP, Phaeophyta; RP, Rhodophyta; EP, Eustigmatophytes; RH, Raphidophyta; DT, Diatoms; PT, plastid; GP, Glaucophyta; CA, Charophyta; CR, Chromerida; HP, Haptophyta; APIC, apicoplast.

a Percentage of the reference genome sequence that is covered by one or more reads in the mapping contig.

b Percentage of the coding region (tRNA-, rRNA-, and protein-coding genes) in the organelle genome. The “% coding” of each genome was determined for this study using the function “extract annotation” in Geneious. We extracted tRNA-, rRNA-, and protein-coding (coding sequence) gene annotations, then excluded spurious annotations and calculated the final length of coding sequences altogether.

Indeed, for each of the species and genomes we explored, the raw RNA-seq reads covered the entire or nearly entire organelle DNA, regardless of taxonomic grouping, organelle type (*i.e.*, mtDNA *vs.* ptDNA), or underlying genomic architecture (Figure 1, Figure S1, Table 1, and Table S1). Not only was the overall read coverage high across the various mitochondrial and plastid genomes (85–100%), but the mean read depth (reads/nt), with few exceptions, was consistently high, ranging from 5 to >23,000 (Table 1). Assuming the RNA-seq reads that mapped correspond to *bona fide* organelle-derived transcripts (see below), these findings suggest that transcription is pervasive, spanning most or all of the organelle genome, including noncoding regions, in a diversity of plastid-bearing protists.

Close inspection of the RNA-seq mapping results revealed some interesting trends within and among the various lineages and genomes (Figure 2, Figure 3, and Figure 4). As expected, the overall RNA read coverage was particularly high (93–100% of the reference genome) for the miniature and highly compact mtDNAs of the five apicomplexan parasites in our dataset (Figure 2), and when applicable (*e.g.*, *Babesia bovis*) it extended into and encompassed the entire mitochondrial telomeres, as has been observed for linear mtDNAs from other lineages (Tian and Smith 2016). These results are consistent with earlier work on apicomplexans showing that their mitochondrial genomes are transcribed in a polycistronic manner (Ji *et al.* 1996; Rehkopf *et al.* 2000), and reinforce the notion that mitochondrial telomeres are involved in gene expression.

The RNA-seq data of the circular-mapping mtDNAs from the green alga *Chlamydomonas moewusii*, the glaucophyte alga *Cyanophora paradoxa*, and the stramenopile alga *Heterosigma akashiwo* are also consistent with a polycistronic mode of transcription, revealing deep, genome-wide RNA coverage across most of the chromosomes, including intergenic regions (Figure 3). Full transcription also appears to be occurring in the mtDNAs from other major algal groups, including brown algae (*e.g.*, *Fucus vesiculosus*), red algae (*e.g.*, *Porphyra purpurea*), dinoflagellate algae (*e.g.*, *Symbiodinium minutum*), and diatom algae (*e.g.*, *Pseudo-nitzschia multiseries*), as well as in both compact and moderately bloated mtDNAs (57–90% coding) (Figure S1, Table 1, and Table S1).

Almost identical trends were observed for the plastid genome data, all of which showed 85.5–100% RNA coverage and a mean read depth of 72–5524 per nucleotide (Figure 4 and Table 1). Like with the mtDNAs, the overall RNA-seq read coverage was especially high for small, compact ptDNAs, such as those from apicomplexan parasites (*e.g.*, *Toxoplasma gondii*) (Table 1) and that of the nonphotosynthetic green alga *Helicosporidium* sp. (∼37 kb; ∼95% coding), 98% of which was represented at the RNA level (Figure 4). The secondary, red algal-derived plastid genomes of the photosynthetic chromerid *Vitrella brassicaformis* and the haptophyte *Emiliana huxleyi* were also well represented in the RNA reads (100 and 97% coverage, respectively, Figure 4), as were those of *C. moewusii* and *H. akashiwo* (Figure S1, Table 1, and Table S1). Overall, these data, alongside previous experiments (Mercer *et al.* 2011; Zhelyazkova *et al.* 2012; Shoguchi *et al.* 2015; Shi *et al.* 2016; Tian and Smith 2016), show that pervasive polycistronic transcription is the norm rather than the exception among mtDNAs and ptDNAs, and underscore the usefulness of RNA-seq for recovering whole-organelle genomes, which can then be used in an array of downstream applications, such as for phylogenetic analyses, barcoding, or measuring nucleotide diversity within and among populations.

### RNA-seq: an untapped resource for organelle research

None of the RNA-seq datasets employed here were initially generated with the intent of studying organelle transcription, and to the best of our knowledge we are the first group to mine organelle transcripts from these experiments. Most, if not all, of the NGS data used here were produced for investigating nuclear gene expression. For instance, the stramenopile alga *Nannochloropsis oceanica* is a model candidate for harvesting biofuels and, thus, the currently available RNA-seq experiments for this species are aimed at better understanding its growth and lipid production, and maximizing its economic potential (Li *et al.* 2014). The same can be said for many of the other species we investigated, such as the seaweeds *Undaria pinnatifida* and *Saccharina japonica*, which are harvested for food (Shan *et al.* 2015, Ye *et al.* 2015), and the apicomplexans *Babesia* spp. and *Theileria* spp., which parasitize livestock (Gardner *et al.* 2005; Brayton *et al.* 2007).

Most scientists do not have the time, resources, or expertise to explore every aspect of an NGS dataset, especially when considering the prodigious amount of information that can be contained within one. But if more scientists knew how easy it was to mine organelle transcriptomes from RNA-seq data, they might be more inclined to study various aspects of organelle genetics, even if it was merely collecting a few sequences for building a phylogenetic tree or for barcoding (Smith 2013). And one cannot forget that organelle biology is intimately tied to that of the nucleus; to fully understand the latter one needs to study the former, and vice versa (Woodson and Chory 2008).

As shown here, and elsewhere (Shi *et al.* 2016; Tian and Smith 2016), complete organelle genomes can be easily and quickly reconstructed from NGS experiments, provided that these experiments were generated in a way that did not exclude organelle transcripts from the sequencing libraries. In some instances, only a single RNA-seq dataset was needed to successfully recover an entire organelle transcriptome; we recovered 99.4% of the *Pavlova lutheri* plastid genome from one 6.7 Gb paired-end RNA-seq experiment. In other cases, we had to source multiple transcriptomic experiments to recover the complete organelle genome (Table S1), suggesting that the libraries used for the cDNA sequencing were depauperate in organelle-derived transcripts. This could be because RNA-seq libraries are often filtered for polyadenylated transcripts (mRNA) and in some lineages organelle RNA can become unstable upon polyadenylation (Rorbach *et al.* 2014). However, other library preparation techniques are much more organelle friendly, including those that target noncoding nuclear RNAs (Di *et al.* 2014) as well as those catered to total cellular RNA (Hotto *et al.* 2011).

One must be careful not to overstate or exaggerate the usefulness of online RNA-seq data for organelle research. There are limitations to what can be deduced about gene expression from the mapping or *de novo* assembly of sequencing reads. Moreover, NGS data downloaded from public databanks can have little or no accompanying information about how they were generated, leaving users guessing about the underlying experimental conditions. And this is to say nothing about the problems of combining and comparing RNA-seq data that were generated by different laboratory groups and/or using different protocols. These factors prevented us from carrying out experiments comparing the mapping rates among datasets with different RNA-selection protocols (*e.g.*, poly-A *vs.* rRNA depletion). There is also a danger of confusing the transcripts of nuclear mitochondrial-like sequences and nuclear plastid-like sequences for genuine organelle RNA, but this is less of an issue for protists than it is for animals and land plants (Smith *et al.* 2011). Finally, there is always the possibility of genomic DNA contamination within the cDNA library, even after multiple rounds of DNase treatment (Haas *et al.* 2012), but this is an issue affecting all types of RNA-seq analyses, not just those exploring organelle RNA.

Despite these drawbacks, scouring RNA-seq databases can reveal important features about organelle transcriptional architecture, such as splice variants, post-transcriptional processing, and RNA editing (Castandet *et al.* 2016) — or the absence of such features. For example, there were no signs of substitutional or insertion/deletion RNA editing in any of the organelle genomes we investigated, but we did detect putative polycistronic processing sites (Figure 3 and Figure 4). RNA-seq has also helped identify transcriptional start sites in the plastid genome of barley (Zhelyazkova *et al.* 2012) and whole-genome transcription in land plant ptDNAs (Shi *et al.* 2016). Although not employed in this study, differential (d)RNA-seq and strand-specific (ss)RNA-seq can provide an even deeper resolution of organelle transcription, exposing antisense RNAs and small noncoding RNAs (Mercer *et al.* 2011; Zhelyazkova *et al.* 2012). As more dRNA-seq and ssRNA-seq experiments are deposited in the SRA (mostly from model species), they can be used to examine fine-tuned features of organelle gene expression following a similar approach to that taken here.

An emerging and recurring theme from organelle transcriptional studies (including this one) is that mitochondrial and plastid genomes are pervasively transcribed (Mercer *et al.* 2011; Zhelyazkova *et al.* 2012; Dietrich *et al.* 2015; Shoguchi *et al.* 2015; Shi *et al.* 2016; Tian and Smith 2016). This is also true for the genomes of alphaproteobacteria and cyanobacteria (Landt *et al.* 2008; Schlüter *et al.* 2010; Mitschke *et al.* 2011a,b; Shi *et al.* 2016), suggesting that pervasive organelle transcription is an ancestral trait passed down from the bacterial progenitors of the mitochondrion and plastid (Shi *et al.* 2016). Many nuclear genomes also show pervasive transcription (Berretta and Morillon 2009), including those of *Saccharomyces cerevisiae* (David *et al.* 2006), *Drosophila melanogaster* (Stolc *et al.* 2004), *Oryza sativa* (Li *et al.* 2006), and *Mus musculus* (Carninci *et al.* 2005). It is estimated that up to ∼75% of the human nuclear genome can be transcriptionally active when looking across tissues and subcellular compartments (Djebali *et al.* 2012). In fact, the more we study genome-wide transcription, the more we realize that few regions in a genome are entirely exempt from transcription and that genomes are veritable “RNA machines,” producing multiple types of RNA from end to end (Amaral *et al.* 2008; Wade and Grainger 2014). Some have suggested that pervasive transcription can provide raw RNA material for new regulatory pathways (Libri 2015). However, certain bacteria can repress pervasive transcription (Lasa *et al.* 2011; Singh *et al.* 2014), so obviously it is not a good strategy all of the time, at least in some systems.

It remains to be seen if big (>>100 kb) organelle genomes, such as land plant mtDNAs (Sloan *et al.* 2012) and chlamydomonadalean ptDNAs (Featherston *et al.* 2016), are fully transcribed, but preliminary work suggests that they are. RNA-seq analyses revealed complete transcription of the *Symbiodinium minutum* mtDNA (∼327 kb) (Shoguchi *et al.* 2015), *Chlamydomonas reinhardtii* ptDNA (∼204 kb), and other bloated organelle DNAs (Shi *et al.* 2016). Therefore, unraveling pervasive transcription in small and giant organelle genomes across eukaryotes could indicate that noncoding organelle RNAs actually have important, undescribed functions. One should be careful not to mistake transcription for function (Doolittle 2013) and not to underestimate transcriptional noise (Struhl 2007), but noncoding organelle RNAs (both long and short) are known to carry out crucial regulatory functions (Hotto *et al.* 2011; Small *et al.* 2013; Dietrich *et al.* 2015). Perhaps having more noncoding DNA and therefore more noncoding RNA leads to increased regulatory control of certain metabolic pathways within organelles [*e.g.*, those for the development of different plastids in land plants (Jarvis and López-Juez 2013)] or more fine-tuned responses to environmental conditions [*e.g.*, changing trophic strategies in mixotrophic algae (Worden *et al.* 2015)]. But if so, why is there such a massive variation in organelle genome size (and transcriptome size) within and among lineages (Khaitovich *et al.* 2004; Lynch *et al.* 2006; Smith and Keeling 2015; Smith 2016a; Figueroa-Martinez *et al.* 2017a,b)? Alas, there is still a lot to be learned about organelle gene expression, and thankfully online RNA-seq data are here to help pave the way.

### Conclusions

The primary goal of this study was to show that entire organelle genome sequences from diverse plastid-containing species can be reconstructed from publicly available RNA-seq datasets within the SRA, as has been previously argued (Smith 2013). On this front, we were successful: algal mtDNAs and ptDNAs from disparate lineages consistently undergo full or nearly full transcription. Thus, available RNA-seq data are an excellent starting point and an untapped resource for exploring transcriptomic and genomic architecture from poorly studied species. Nevertheless, online RNA-seq experiments have their limitations and drawbacks, and one should be mindful when employing such data. It will be interesting to see if the major trends reported here will be borne out by future investigations, specifically those of larger organelle genomes. Ultimately, a deep understanding of organelle gene expression requires a multi-pronged approach, employing both traditional molecular biology techniques as well as more modern high-throughput methods (Sanita Limá *et al.* 2016).

## Supplementary Material

Supplemental material is available online at www.g3journal.org/lookup/suppl/doi:10.1534/g3.117.300290/-/DC1.
