# Supplementary figures and images for: Pervasive, Genome-Wide Transcription in the Organelle Genomes of Diverse Plastid-Bearing Protists
Source: G3 (Bethesda). 2017 Sep 20;7(11):3789–96. doi: 10.1534/g3.117.300290 (PMC5677165; doi:10.1534/g3.117.300290)

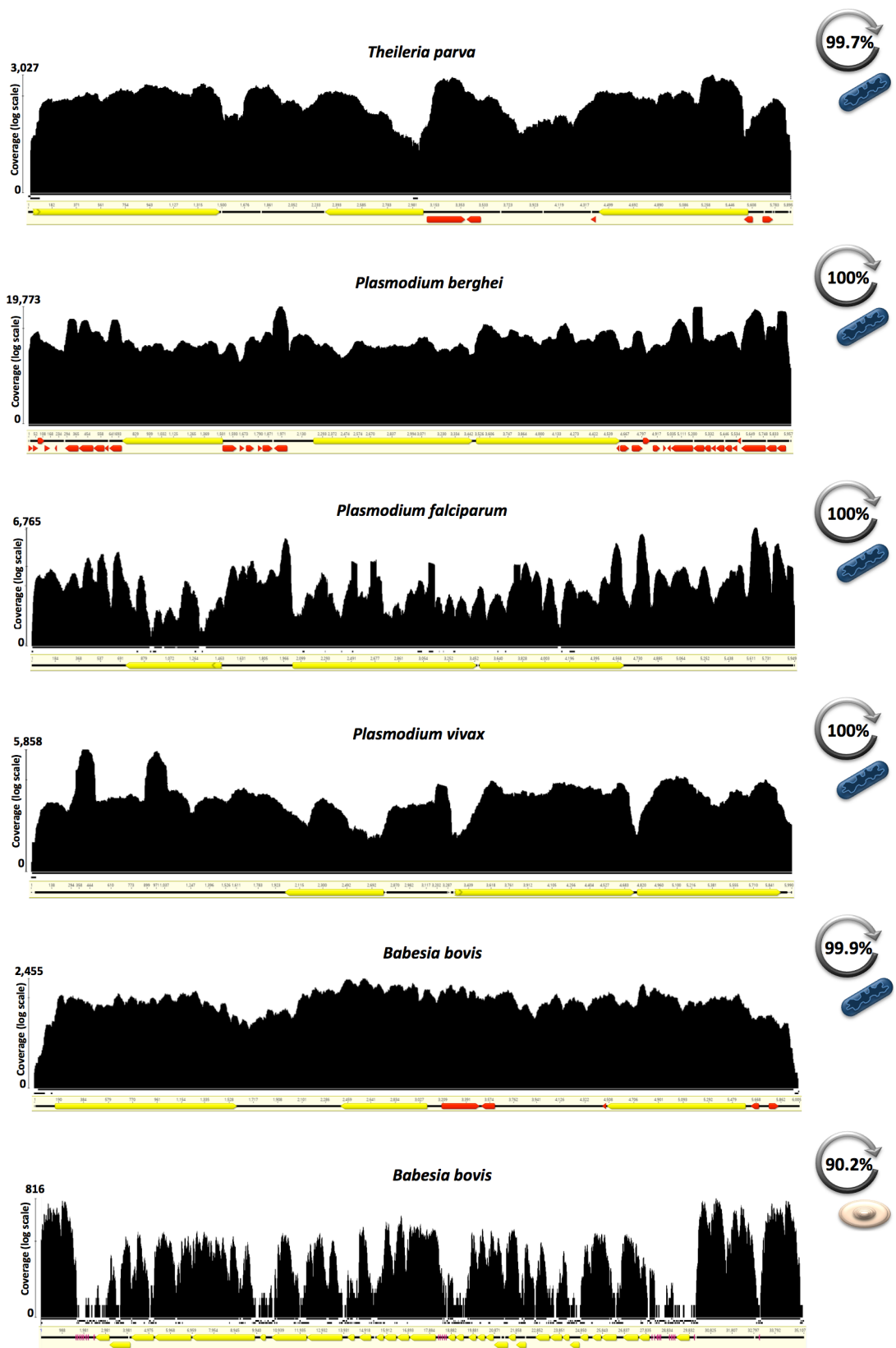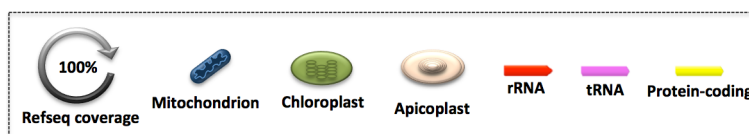

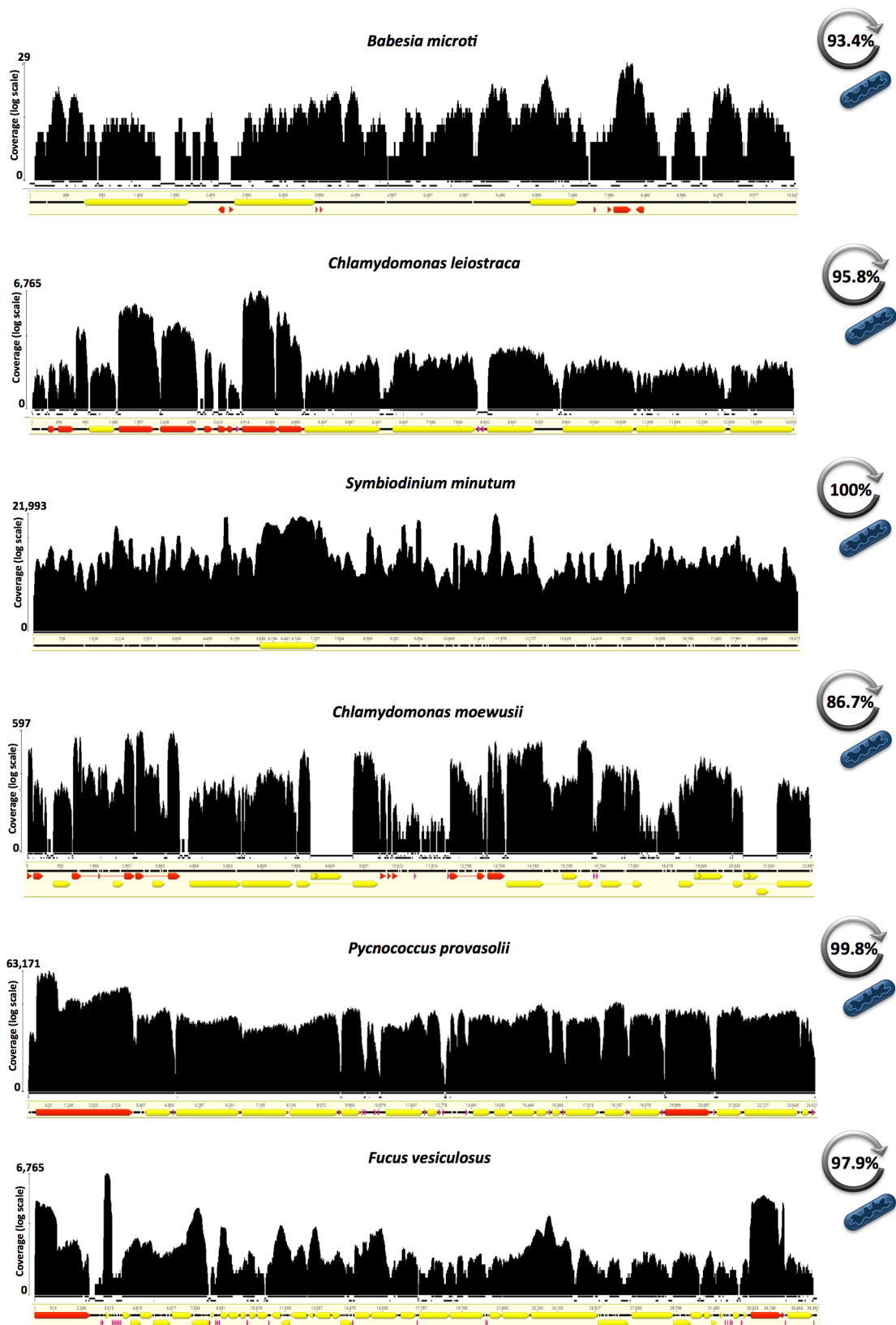

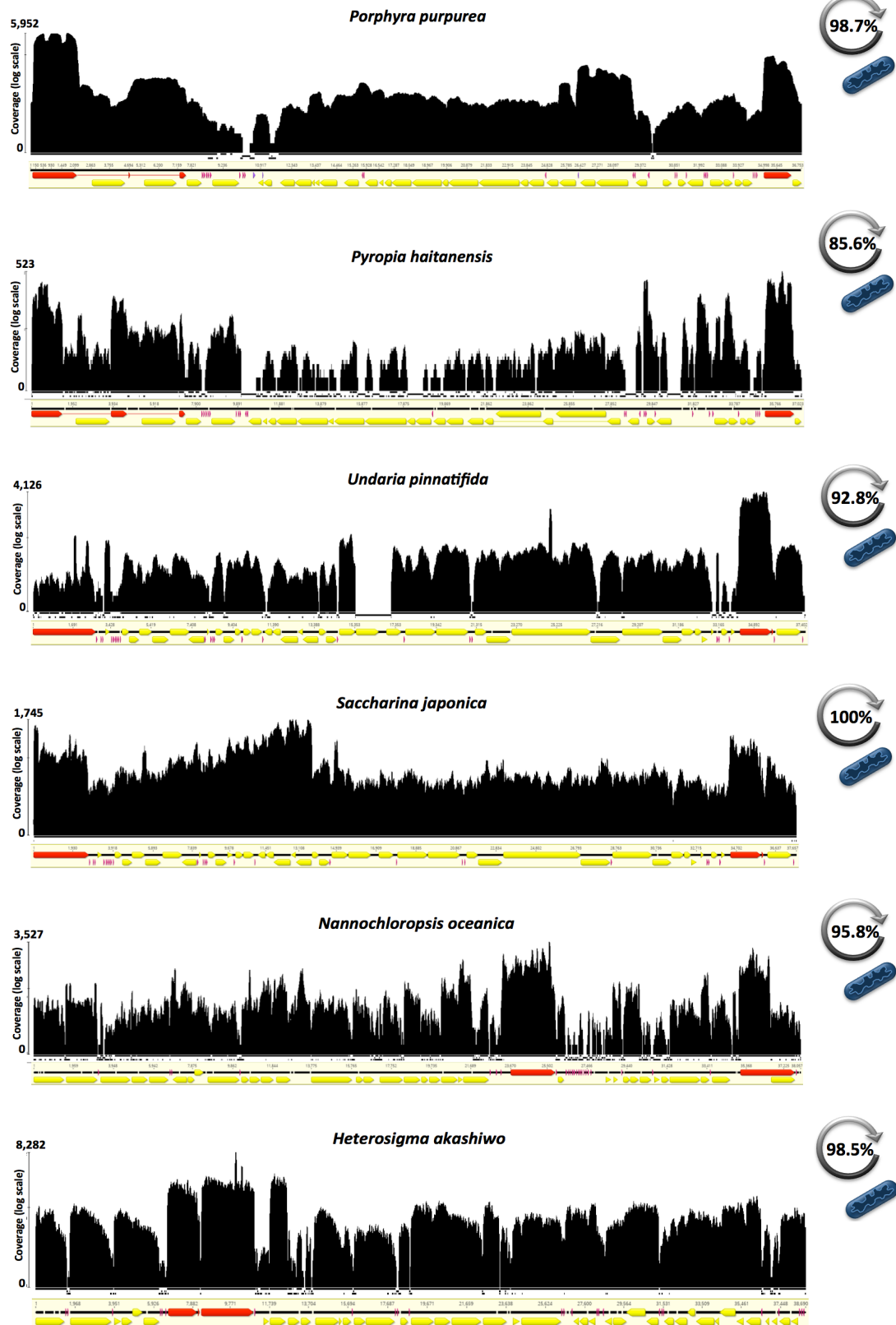

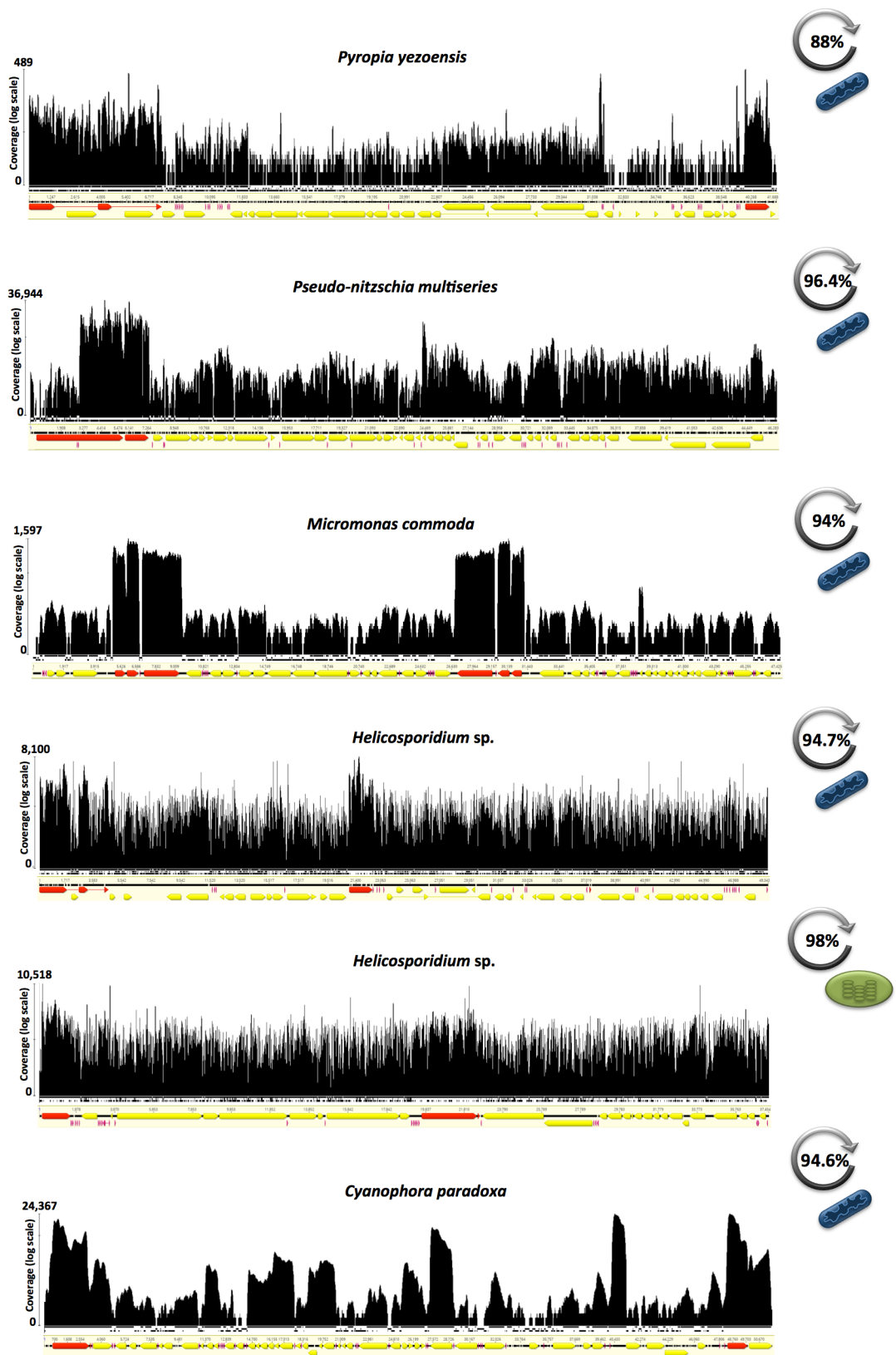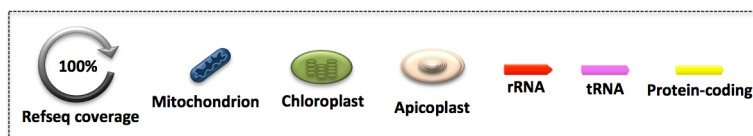

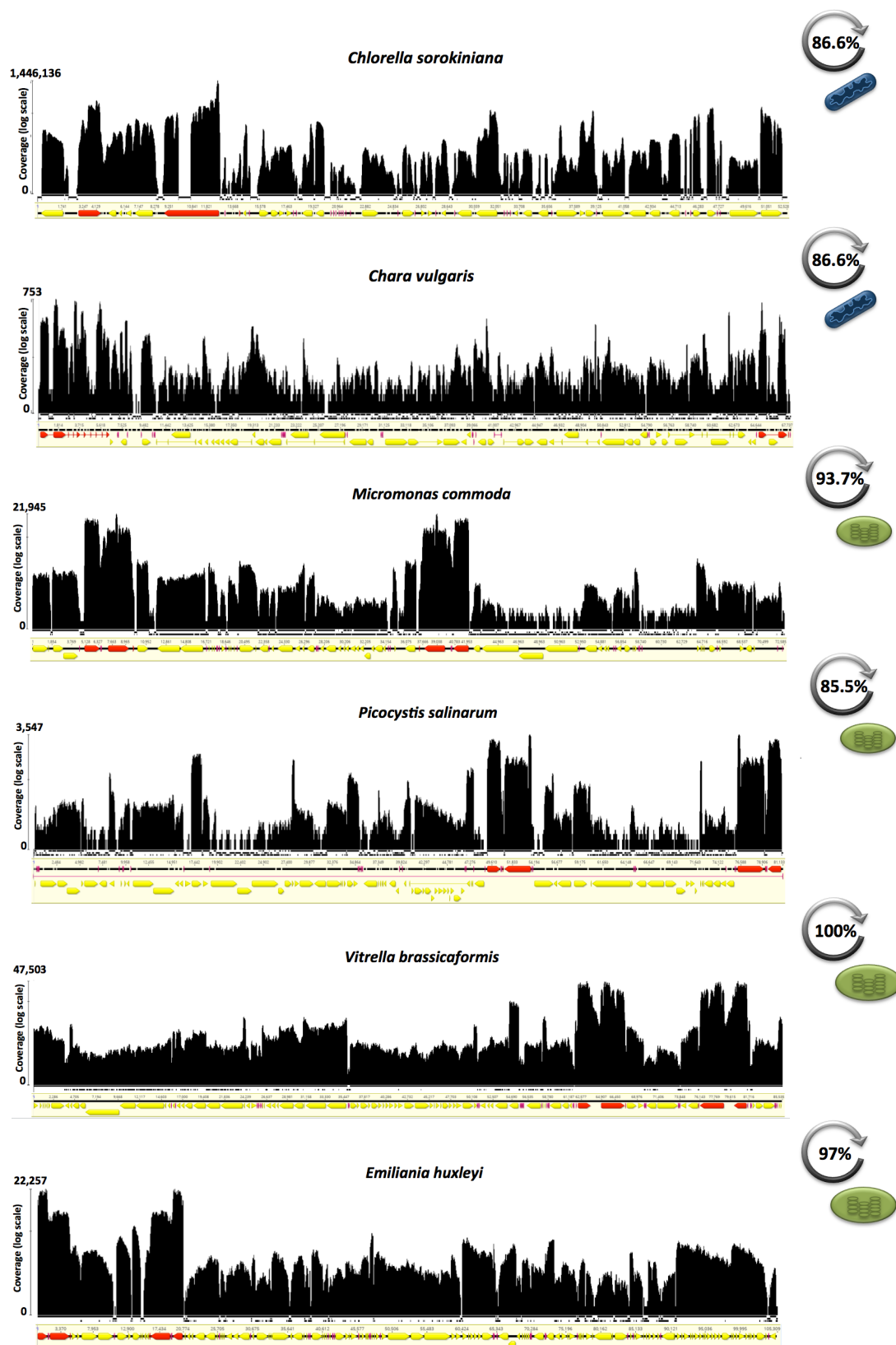

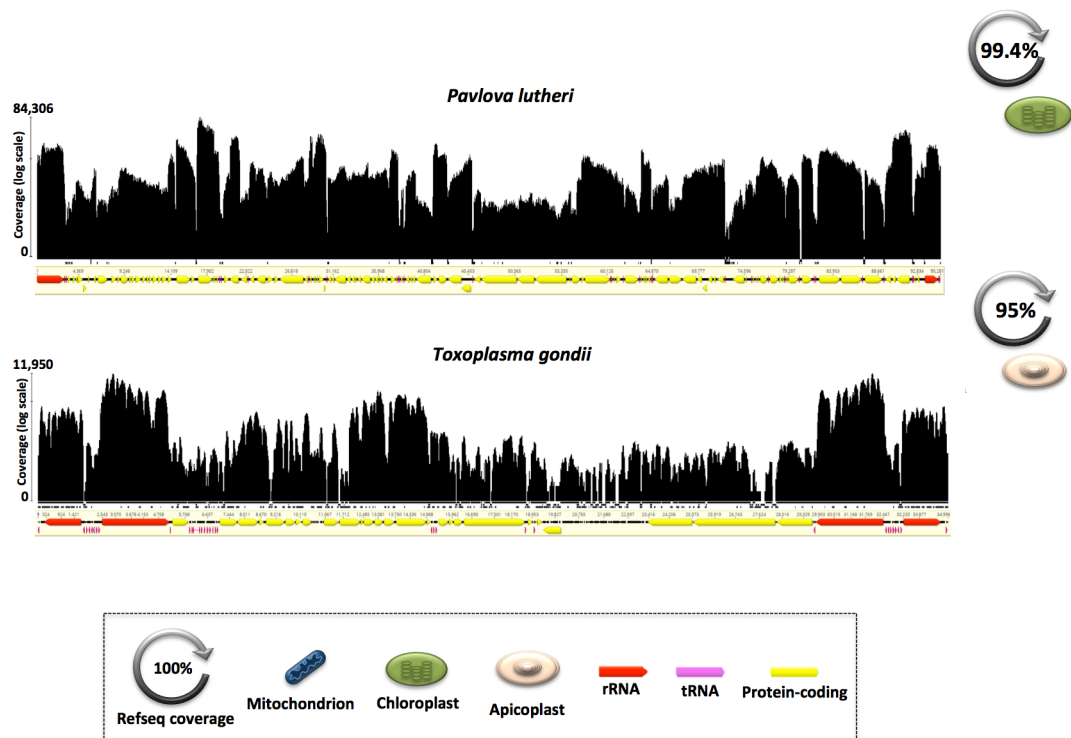

**Figure S1** Transcription maps for all 30 species analysed.

Supplement: Supplementary file 1 [file 3789FigureS1.pdf]
